# Regulation of α-Transducin and α-Gustducin Expression by a High Protein Diet in the Pig Gastrointestinal Tract

**DOI:** 10.1371/journal.pone.0148954

**Published:** 2016-02-12

**Authors:** Roberto De Giorgio, Maurizio Mazzoni, Claudia Vallorani, Rocco Latorre, Cristiano Bombardi, Maria Laura Bacci, Monica Forni, Mirella Falconi, Catia Sternini, Paolo Clavenzani

**Affiliations:** 1 Department of Medical and Surgical Sciences, University of Bologna, Bologna, Italy; 2 Department of Veterinary Medical Sciences, University of Bologna, Bologna, Italy; 3 Department of Biomedical and Neuromotor Sciences, University of Bologna, Bologna, Italy; 4 CURE Digestive Diseases Research Center, Digestive Diseases Division, Department of Medicine, David Geffen School of Medicine, University of California Los Angeles, Los Angeles, California, United States of America; 5 Department of Neurobiology, David Geffen School of Medicine, University of California Los Angeles, Los Angeles, California, United States of America; 6 Veterans Administration, Greater Los Angeles Health system, Los Angeles, California, United States of America; National Institute of Agronomic Research, FRANCE

## Abstract

**Background:**

The expression of taste receptors (TASRs) and their signalling molecules in the gastrointestinal (GI) epithelial cells, including enteroendocrine cells (EECs), suggests they participate in chemosensing mechanisms influencing GI physiology via the release of endocrine messengers. TASRs mediate gustatory signalling by interacting with different transducers, including α-gustducin (G_αgust_) and α-transducin (G_αtran_) G protein subunits. This study tested whether G_αtran_ and G_αgust_ immunoreactive (-IR) cells are affected by a short-term (3 days) and long-term (30 days) high protein (Hp) diet in the pig GI tract.

**Result:**

In the stomach, G_αgust_ and G_αtran_-IR cells contained serotonin (5-HT) and ghrelin (GHR), while in the small and large intestine, G_αgust_ and G_αtran_-IR colocalized with 5-HT-, cholecystokinin (CCK)- and peptide YY (PYY)-IR. There was a significant increase in the density of G_αtran_-IR cells in the pyloric mucosa in both short- and long-term Hp diet groups (Hp3 and Hp30) vs. the control group (Ctr) (*P*<0.05), while the increase of G_αgust_-IR cells in the pyloric mucosa was significant in Hp30 group vs. Ctr and vs. Hp3 (*P*<0.05); these cells included G_αtran_ / 5HT-IR and G_αtran_ / GHR-IR cells (*P*<0.05 and *P*<0.001 vs. Ctr, respectively) as well as G_αgust_ /5-HT-IR or G_αgust_ / GHR-IR cells (*P*<0.05 and *P*<0.01 vs. Ctr, respectively). In the small intestine, we recorded a significant increase in G_αtran_-IR cells in the duodenal crypts and a significant increase of G_αgust_-IR cells in the jejunal crypts in Hp3 group compared to HP30 (*P*<0.05). With regard to the number of G_αtran_-G_αgust_ IR cells colocalized with CCK or 5-HT, there was only a significant increase of G_αtran_ / CCK-IR cells in Hp3 group compared to Ctr (*P* = 0.01).

**Conclusion:**

This study showed an upregulation of selected subpopulations of G_αgust_ / G_αtran_-IR cells in distinct regions of the pig GI tract by short- and long-term Hp diet lending support to TASR-mediated effects in metabolic homeostasis and satiety mechanisms.

## Introduction

The gastrointestinal (GI) tract has the important task of food digestion followed by absorption and metabolism of nutrients such as amino acids, sugars and fatty acids. These food-derived components are detected by a “nutrient chemosensing system” involving luminal sensors in the GI mucosa [1–6], which send information to the nervous system to initiate physiological responses regulating food intake and eating behaviour through the gut-brain axis [1–3,6]. The identification of taste receptors (TASRs) and their signalling molecules along the mammalian GI tract and the observation that TASR ligands in the gut lumen induce functional responses such as activation of vagal afferents, alteration of food intake and GI motility, aversion, release of peptides and regulation of glucose homeostasis [7–13], support a key role of these receptors in the luminal chemosensing processes. In the GI tract, TASRs are expressed by epithelial cells, mainly enteroendocrine cells (EECs). Their stimulation *in vivo* and *in vitro* initiates a signalling cascade that ultimately leads to release of chemical messengers [8,11,14]. This mechanism has been postulated to activate neural reflex pathways including intrinsic and extrinsic neurons affecting gut physiology and energy homeostasis [1–3,6].

TASRs are G-protein coupled receptors comprising two major families: the TAS1Rs family composed by three receptors (TAS1R1, TAS1R2 and TAS1R3) that function as dimers to detect umami (TAS1R1 with TAS1R3) and sweet (T1R2 with TAS1R3) [15–17], and a large family of TAS2Rs (about 25 subtypes in humans and >30 in rodents) that detect an array of diverse bitter compounds [18,19]. Upon activation, TASRs coupled to G-protein related signalling messengers, α-gustducin (G_αgust_), α-transducin (G_αtran_), and other transducers as well, lead to the intracellular Ca^2+^ increase and cellular response. G_αgust_ and G_αtran_ have been identified throughout the digestive system, from the tongue down to the distal part of the GI tract of different mammalian and non-mammalian species [13,20–29]. The involvement of different G protein subunits, such as G_αgust_, G_αtran_ and other Gi-family alpha subunits, in taste transmission has been demonstrated by several findings including the observations that not all taste cells contain G_αgust_, that gustatory transduction was not completely abolished in mice with deletion of *G*_αgust_ gene, and that G_αtran_ can partially rescue the taste response in these mice [30–32].

In our previous studies, we found that the TASR-related G protein subunits, G_αgust_ and G_αtran_ in the gut are regulated by different diet manipulation, including fasting and refeeding, high-fat diet and a low cholesterol mimicking diet in the mouse and porcine gut [27, 28]. These findings suggest plasticity in taste-related molecules in the GI tract in response to different feeding states and caloric intake. Increasing evidence support the notion that high protein diets reduce food intake, facilitate weight loss, and improve body composition in both humans and animal models [33–35]. Thus, our study was designed to test whether short- and long-term high protein (Hp) diet affected the expression of G_αgust_ and G_αtran_ immunoreactive (IR) cells throughout the pig GI tract. In addition, we characterized the phenotype of G_αgust_ -and G_αtran_-IR cells with special emphasis on chemical messengers such as peptides and biogenic amines involved in satiation and body weight regulation.

## Materials and Methods

### Animals

The experiments were performed at the Physiology unit of the Department of Veterinary Medical Sciences of the University of Bologna. The study was conducted according to relevant national and international guidelines on Animal Experimentations. The procedure was reviewed and approved in advance by the Scientific Ethics Committee for Animal Experimentation of the University of Bologna and by the Italian Ministry of Public Health. Twelve Large White/Duroc hybrid female pigs (12 weeks old, live weight 33.6 ± 3.05 kg) were purchased from a commercial breeder. Upon arrival, pigs were weighed, clinically examined and arranged in multiple boxes (n = 4 in each box) with slatted floor, previously cleaned and sanitized by an authorized operator. They were immediately fed with standard diet containing 14.5% protein (Big 30 Flour, Cooperativa Agricola Tre Spighe, Castel Guelfo, Bologna, Italy); tap water was freely available. The clinic exam ensured all the animals were healthy and did not show pathologies that could interfere with the experimental results. All pigs were fed with standard diet for 2 weeks in order to allow the normalization of GI function. Animals were then randomly assigned to three experimental groups; one group (n = 4) received standard diet and served as control (Ctr); one group (n = 4) was fed high protein diet (35% protein) for 3 days (Hp3); and one group (n = 4) was fed high protein diet (35% protein) for 30 days (Hp30). The component of experimental diet, energy density as well as body weight and food consumption are described in S1 and S2 Tables. Feeding behaviour was recorded every week and pigs were weighed at the beginning and at the end of the experimental design (i.e., at 0, 3 and 30 days). At the end of the experiment, animals were euthanized with i.v. bolus of Tanax (embutramide, mebenzonio iodure, tetracaine) (10 mL / head; Intervet Italia Srl, Milan, Italy) after premedication with i.m. azaperone 3 mg / kg (Stresnil; Janssen-Cilag SpA, Milan, Italy) and surgical anaesthesia, induced with 20 mg/kg ketamine i.m. (Ketavet 100; Intervet Italia Srl, Milan, Italy) and with an i.v. bolus of sodium thiopental (300 mg / animal, Pentothal Sodium; Intervet Italia Srl, Milan, Italy). We euthanized two animals for the Ctr group at 3 days and two at 30 days since in preliminary studies (data not shown) we did not see significant differences of mucosal morphology.

### Samples collection

Specimens of the GI tract included stomach mucosa (cardiac, near the gastric diverticulum; pyloric, close to the pyloric sphincter), duodenum (about 10 cm from the pyloric sphincter), middle jejunum, ileum, cecum, ascending colon (near the centrifugal turns), descending colon (about 25 cm from the anus) and rectum (in the *ampulla recti*). Specimens were pinned flat on balsa wood, fixed in 4% buffered paraformaldehyde / 0.1 M phosphate buffer, pH 7.4 for 48 h at 4°C, dehydrated and embedded in paraffin [27].

### Immunohistochemistry

Serial (5 μm thick) sections mounted on poly-L-lysine-coated slides were subjected to single and double immunofluorescence staining using antibodies directed to G_αtran_ or G_αgust_ and specific EEC subtype markers such as ghrelin (GHR), gastrin/cholecystokinin (GAS/CCK), 5-hydroxytryptamine (5-HT), peptide YY (PYY) shown in Table 1. Briefly, sections were deparaffinized with xylene, rehydrated with graded ethanol, and heat-treated in a microwave (2 cycles at 750 W, 5 min each) in sodium citrate buffer (pH 6.0) to retrieve the antigenicity. Sections were incubated in 10% appropriate normal serum in 0.01 M phosphate buffer saline (PBS) (1 h at room temperature) to prevent non-specific bindings, and subsequently incubated overnight with primary antibodies diluted in PBS and 5% of normal serum. After primary antibody incubation, a mixture of fluorescein isothiocyanate (FITC)-conjugated, tetramethyl rhodamine isothiocyanate (TRITC)-conjugated, Alexa Fluor^®^ 594- and Alexa Fluor^®^ 488-conjugated secondary antibodies diluted in PBS (Table 1) was added for 1 h at room temperature. Finally, the slides were washed in PBS and cover-slipped with buffered glycerol, pH 8.6.

**Table 1:** List and dilutions of primary and secondary antibodies.

**Primary antibodies**	**Code**	**Species**	**Dilution**	**Supplier**
α-Transducin	sc-390	rabbit	1:200	Santa Cruz
α-Gustducin	sc-395	rabbit	1:200	Santa Cruz
Cholecystokinin/Gastrin	CCK/GAS # 9303	mouse	1:1000	CURE/DDRC
Ghrelin	sc-10368	goat	1:400	Santa Cruz
5-hydroxitryptamine	ab16007	mouse	1:200	Abcam
Peptide YY	PAB17185	guinea pig	1:1000	Abnova
**Secondary antibodies**	**Code**	**Species**	**Dilution**	**Supplier**
Alexa 594 conjugated anti-mouse IgG	A11005	goat	1:600	Mol. Probes
Alexa 488 conjugated anti-rabbit IgG	A21206	donkey	1:1000	Mol. Probes
FITC conjugated anti-rabbit IgG	401314	goat	1:500	Calbiochem
TRITC conjugated anti-goat IgG	705-295-003	donkey	1:1000	Jackson
TRITC conjugated anti-guinea pig IgG	AP108R	goat	1:100	Chemicon/Millipore

CURE/DDRC (P30DK041301), UCLA, Los Angeles, CA, USA. Chemicon International, Temecula, CA, USA. Abcam, Cambridge, UK. Santa Cruz Biotecnology, Inc., CA, USA. Abnova, Jhouzih St. Neihu District. Taipei City, Taiwan. Calbiochem- Novabiochem Corporation, San Diego, CA, USA. Molecular Probes, Eugene, OR., USA. Jackson ImmunoResearch Laboratories, Inc., West Grove, PA, USA.

### Specificity of antibodies

Specificity of G_αtran_, G_αgust_ and CCK/GAS has been previously demonstrated by Western Blot and/or pre-adsorption test [27]. GHR, 5-HT and PYY antibody specificity was assessed by pre-adsorption with an excess of the homologous peptide (GHR, sc-10368 P, Santa Cruz, CA, USA; 5-HT, H9523, Sigma-Chemicals, St. Louis, MO, USA; and PYY, 059–06, Phoenix Pharm. Inc., Burlingame, CA, USA, respectively) (S1 Fig).

### Cell counting and statistical analysis

Cell counting was performed with a 40X objective lens using a Zeiss Axioplan microscope (Carl Zeiss, Oberkochen, Germany) with appropriate filter cubes. Images were obtained with a Polaroid DMC digital photocamera (Polaroid, Cambridge, Mass., USA), and minimal adjustments to brightness and contrast were made with Corel Photo Paint and Corel Draw (Corel, Dublin, Ireland). Each specimen was evaluated and counted by two investigators in a blind fashion. For each animal, G_αtran_- and G_αgust_-IR cells were counted in 36 random microscope fields (each field 0.28 mm^2^), for a total area of 10 mm^2^, in the cardiac and pyloric mucosa, in 50 randomly selected villi and glands in the small intestine, and in 50 glands in the large intestine. Only villi and glands perpendicular to the *muscularis mucosae* were evaluated. The values obtained from counting G_αtran_- and G_αgust_-IR cells were grouped for each experimental group (Ctr, Hp3 and Hp30) and the means were calculated. Moreover, the mean numbers of cells showing a colocalization of G_αtran_ or G_αgust_-IRs with different EEC markers were calculated. Results were expressed as mean ± standard deviation (SD). Data were analysed by one-way ANOVA (Graph Prism 4, GraphPad Software, Inc., La Jolla, CA, USA). A *P*<0.05 was considered statistically significant.

## Results

### Distribution and neurochemical characterization of G_αtran_-IR and G_αgust_-IR cells in the GI tract

**Fig 1:**
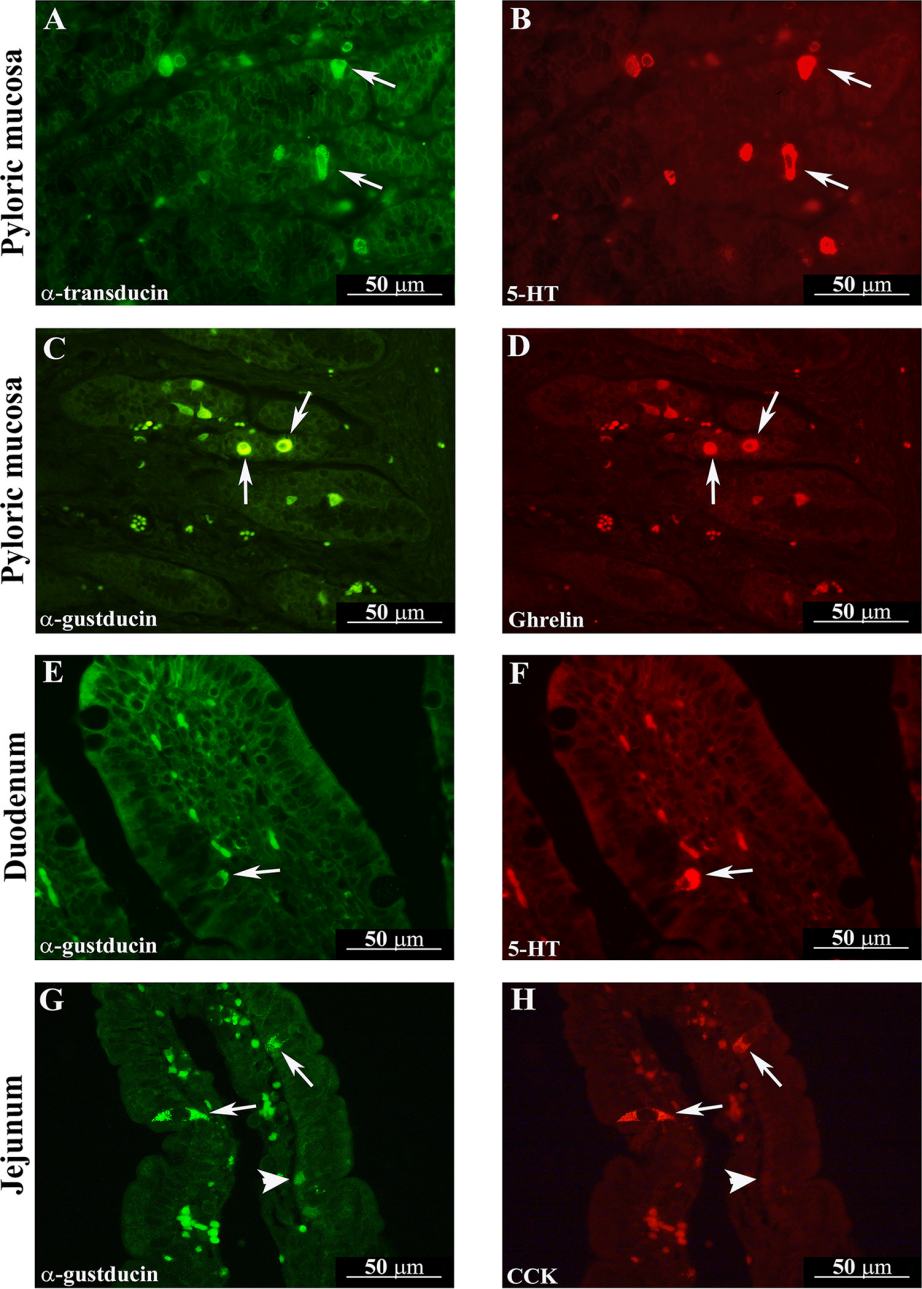
Colocalization of G_αtran_-IR with 5-hydroxytryptamine (5-HT) in the pyloric mucosa following short-term Hp diet (Hp3) (arrows in A and B). The arrows in the photomicrographs (C), (E) and (G) show G_αgust_-IR cells co-expressing ghrelin (D) in the pyloric mucosa of a pig fed a control diet (Ctr), 5-HT (F) in the duodenum of a pig fed long-term Hp diet (Hp30) and cholecystokinin (CCK) (H) in the jejunum of a Hp30 fed pig. The arrowheads in G and H indicate G_αgust_-IR cells not containing CCK-IR. Generally, the G_αtran_ / G_αgust_ labelled cells were found lying close to the basal lamina of the glands (typical closed-type morphology) (A and C, arrows). Frequently, the G_αtran_ / G_αgust_-IR cells are localized in the surface epithelium of the villi (with typical open-type morphology) (E and G, arrows).

**Fig 2:**
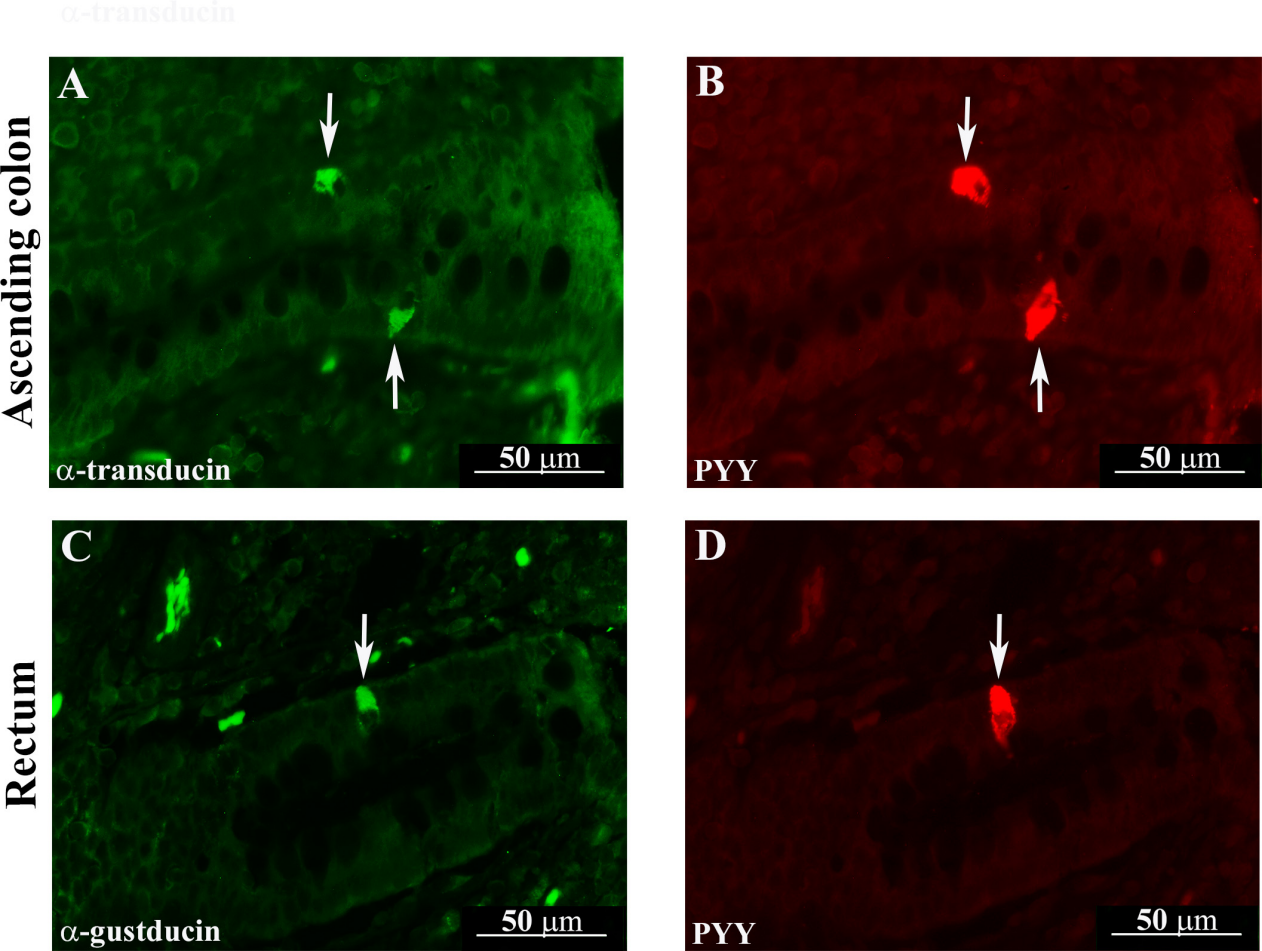
G_αtran_ and G_αgust_ colocalized with peptide YY (PYY) in the ascending colon of an Hp3-fed animal (A and B, arrows) and in rectum of an Hp30-fed pig (C and D, arrows), respectively. G_αtran_ and G_αgust_ positive cells containing PYY-IR located in either the glandular or surface epithelium of the ascending colon and rectum have an open-type morphology.

G_αtran_- and G_αgust_-IR cells were distributed throughout the whole pig GI tract (Fig 1A, 1C, 1E and 1G; Fig 2A and 2C), extending our previous description of G_αtran_-IR cell distribution [27]. Similarly to the distribution of G_αtran_-IR cells as reported in details in our previous publication [27] and confirmed in this study, G_αgust_-IR cells were observed both in the distal third and in the epithelial profile of the gastric mucosa of pyloric region, along the villus-crypt axis of the small intestine, and in the glandular epithelium of the large intestine. Most G_αtran_-and G_αgust_-IR cells had the morphological appearance of “open-type” EECs with an elongated shape, homogenous cytoplasm (Fig 2A and 2C) and two cytoplasmic prolongations, one reaching the lumen and the other the basal lamina (Fig 1G). Other G_αtran_-and G_αgust_-IR cells had the “closed-type” EEC appearance with a round shape without cytoplasmic prolongations (Fig 1A and 1C). Double labelling immunofluorescence showed that the majority of G_αtran_-IR and G_αgust_-IR cells in the cardiac and pyloric mucosa were immunopositive for 5-HT (Fig 1A and 1B). Co-expression of G_αtran_ / 5-HT or G_αgust_ / 5-HT was also observed in the villi and glandular epithelium of the duodenum (Fig 1E and 1F), where some G_αtran_-IR cells were immunopositive for 5-HT, while most G_αgust_ positive cells co-expressed 5-HT. In the cardiac and pyloric mucosa, the majority of G_αtran_-IR and G_αgust_-IR cells co-expressed GHR (Fig 1C and 1D). In the jejunum, most of the G_αtran_- or G_αgust_-IR cells distributed along the crypt-villus axis co-expressed CCK-IR (Fig 1G and 1H). In the large intestine, coexpression of G_αtran_ or G_αgust_ and PYY-IR was seen in elongated cells located in the surface epithelium as well in cells of the glandular epithelium (Fig 2A–2D). The percentages of colocalization of G_αtran_- or G_αgust_-IR cells with EEC subtypes are shown in Tables 2 and 3.

**Table 2:** Percentages of co-localization of G_αtran_- or G_αgust_-IR cells with 5-HT and GHR in the gastric and duodenal mucosa.

Cell types	Cardiac mucosa (%)	Pyloric mucosa (%)	Duodenal villi (%)	Duodenal glands (%)
**G**_**αtran**_ **5HT/ total G**_**αtran**_	95	94	21.2	28.2
**G**_**αgust**_ **5HT/ total G**_**αgust**_	92.2	96.2	95.2	94.6
**G**_**αtran**_ **GHR/ total G**_**αtran**_	82.8	77	————	————
**G**_**αgust**_ **GHR/ total G**_**αgust**_	61.3	74.6	————	————

**Table 3:** Percentages of co-localization of G_αtran_- or G_αgust_-IR cells with CCK- and PYY-IR in the jejunum and large intestine mucosa.

Jejunum (villi) (%)		Jejunum (glands) (%)		Large intestine (%)	
G_αtran_CCK/ total G_αtran_	G_αgust_CCK/ total G_αgust_	G_αtran_CCK/ total G_αtran_	G_αgust_CCK/ total G_αgust_	G_αtran_PYY/ total G_αtran_	G_αgust_PYY/ total G_αgust_
99.8	94.4	96.2	91.1	75	70.2

### Distribution of the G_αtran_- and G_αgust_-IR cells in the three experimental groups (Ctr, Hp3 and Hp30)

**Fig 3:**
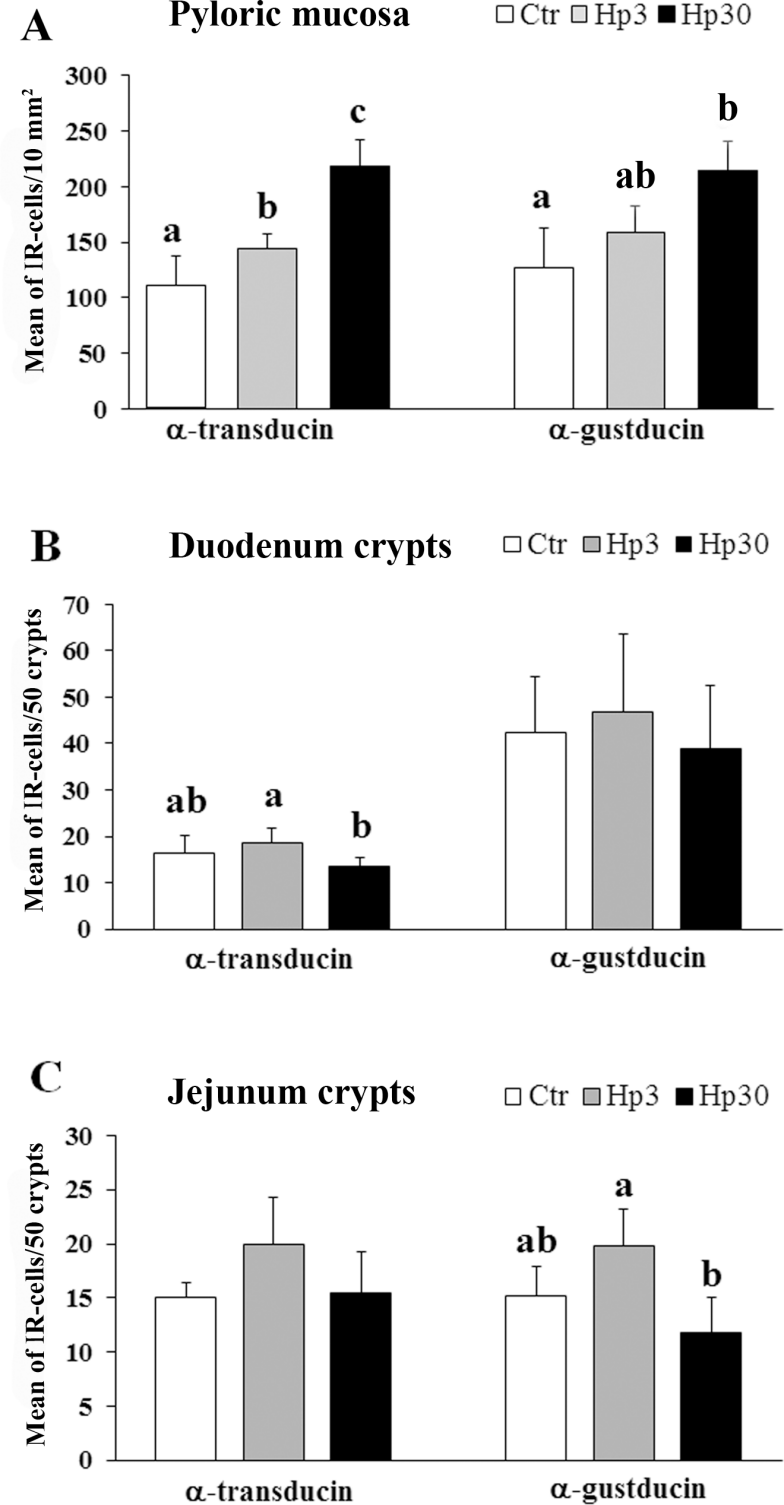
Quantitative assessment of the mean number of G_αtran_ and G_αgust_-IR cells in the pig pyloric mucosa (A), duodenum (B) and jejunum (C) crypts. Different letters indicate a significant (*P*<0.05) statistical difference among groups. Values are expressed as mean ± SD.

In the cardiac mucosa, the density of G_αtran_-or G_αgust_-IR cells was not affected by the administration of the short or long-term Hp diet (Hp3 and Hp30, respectively; not shown). By contrast, in the pyloric mucosa, we observed a significant increase in the density of G_αtran_-IR cells in both Hp3 and Hp30 group compared to Ctr (*P*<0.05) as well as Hp30 vs. Hp3 (*P*<0.05); also, a similar increase was observed for G_αgust-_IR cells in the Hp30 vs. Ctr and Hp3 (*P*<0.05) while there were not a significant increase of G_αgust-_IR cells in Hp3 compared to Ctr (Fig 3A).

In the small intestine, G_αtran_-IR cells were significantly increased in the duodenum crypts in the Hp3 group compared to Hp30, while there was a significant increase of G_αgust_-IR cells in jejunal crypts in Hp3 compared to Hp30 groups (*P*<0.05) (Fig 3B and 3C). Conversely, there were not significant differences between the mean number of G_αtran_- or G_αgust_-IR cells in the duodenal and jejunal villi in the Ctr vs. the different experimental groups (data not shown). In the large intestine, we observed a progressive increase in the number of G_αtran_- or G_αgust_-IR cells from the cecum to the rectum without reaching statistically significant differences among the experimental groups (data not shown).

### Expression of EEC subpopulations of cells in the three experimental groups (Ctr, Hp3 and Hp30)

**Fig 4:**
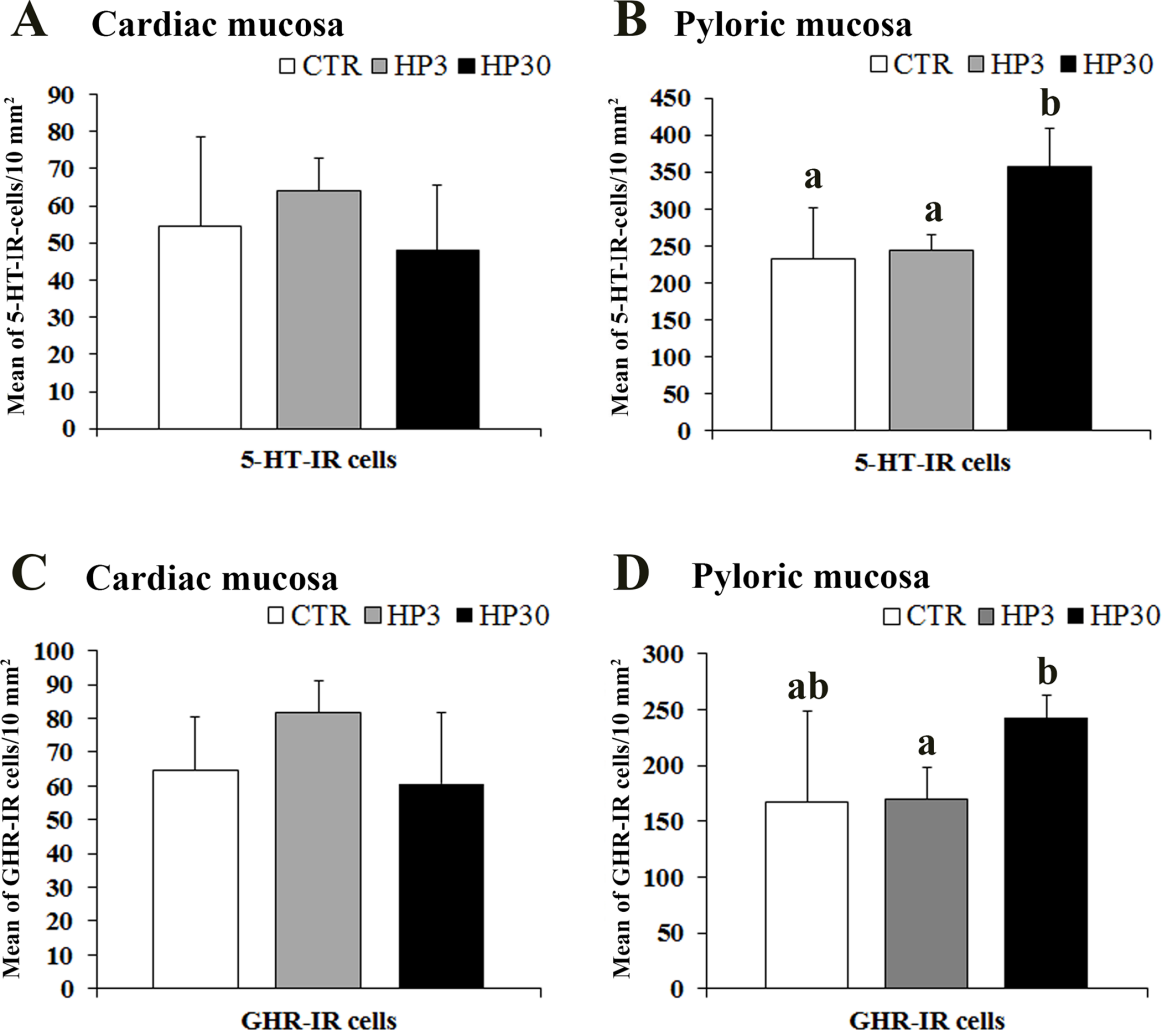
Quantitative assessment of the mean number of 5-HT and GHR-IR cells in the pig cardiac (A and C) and pyloric mucosa (B and D). Different letters indicate a significant (*P*<0.05) statistical difference among groups. Values are expressed as mean ± SD.

In the cardiac mucosa, the number of 5-HT positive cells did not change in the three experimental groups, whereas in the pyloric mucosa, 5-HT-IR cells were significantly more numerous in Hp30 *vs*. Ctr (*P˂*0.01) and *vs*. Hp3 (*P˂*0.01) (Fig 4A and 4B).There were no significant differences in the expression of GHR positive cells among the groups in the cardiac mucosa, while in the pyloric mucosa we observed a significant increase in the mean value of GHR-IR cells in the Hp30 compared to the Hp3 (*P˂*0.01) (Fig 4C and 4D). The density of 5-HT- and CCK-IR cells in the duodenum and jejunum (villi and crypts) did not differ significantly in the three experimental groups. PYY-IR cells were more numerous in the descending colon and rectum *vs*. cecum and ascending colon, but there were no significant differences among the different segments of the large intestine in the three experimental groups.

### Distribution of the different subgroups of G_αtran_- and G_αgust_-IR EECs in Ctr, Hp3 and Hp30 groups

**Fig 5:**
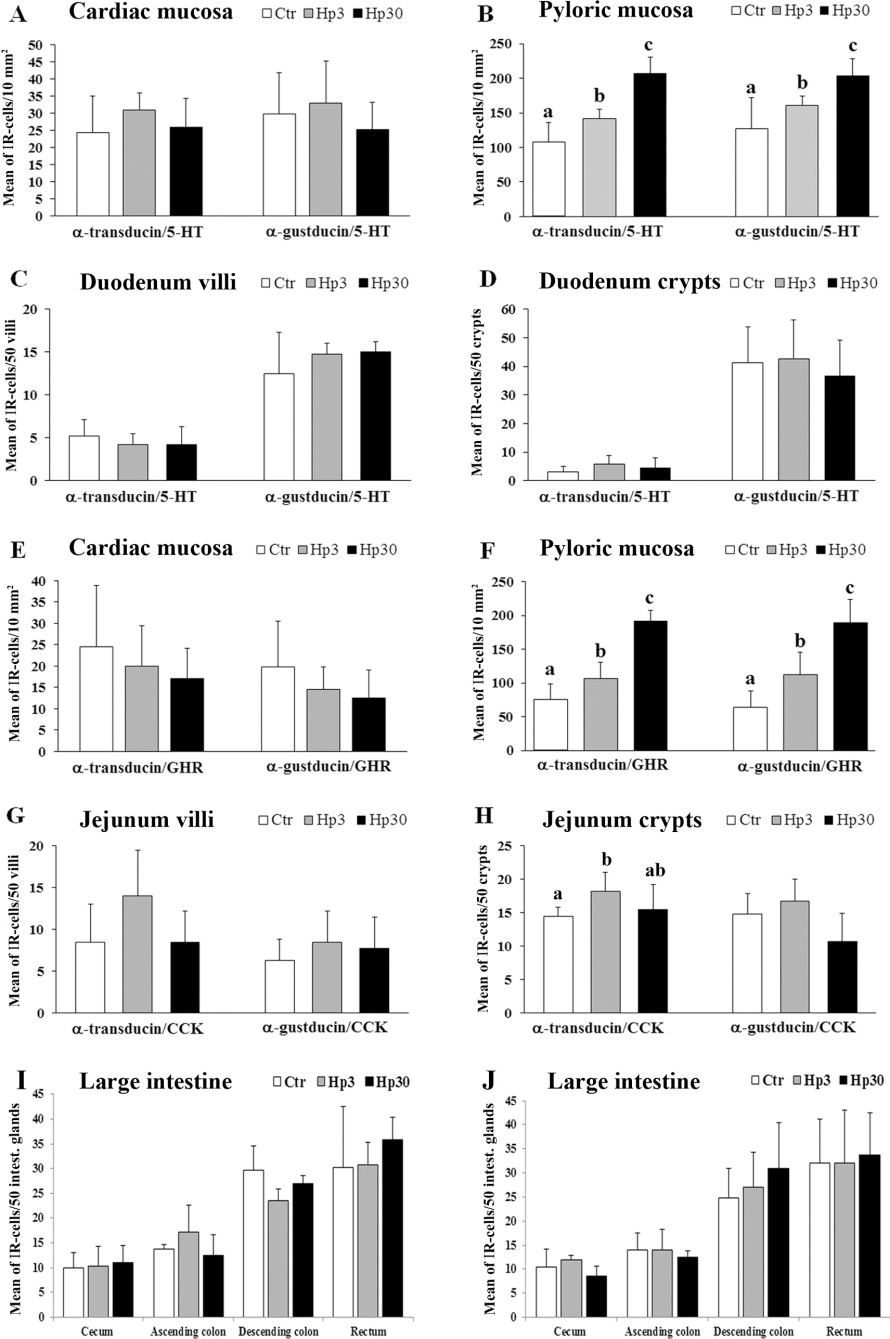
Quantitative evaluation of the mean number of G_αtran_- or G_αgust_ / 5-HT-IR in the cardiac (A) and pyloric mucosa (B), in the villi (C) and crypts (D) of the duodenum. E and F show the mean number of G_αtran_- or G_αgust_ / GHR-IR cells in the pig cardiac and pyloric mucosa, respectively), and in the villi (G) and crypts (H) of the jejunum. Finally, I and J illustrate the mean number of G_αtran_- or G_αgust_ / PYY-IR in the large intestine, respectively. Different letters indicate a significant (*P*<0.05) statistical difference among groups. Values are expressed as mean ± SD.

In the cardiac mucosa, the Hp diet did not produce any significant change in the mean number of G_αtran_ / 5-HT or G_αgust_ / 5-HT-IR cells (Fig 5A), whereas in the pyloric mucosa, the 30-day Hp diet led to a significant (*P*<0.01) increase of G_αtran_ / 5-HT-IR or G_αgust_ / 5-HT-IR cells compared with the others two experimental groups (Ctr and Hp3) (Fig 5B). Moreover, there were no changes in the mean number of G_αtran_ / 5-HT and G_αgust_ / 5-HT cells in Ctr, Hp3 and Hp30 groups both in villi and crypts of the duodenum (Fig 5C and 5D).

The number of cells co-expressing G_αtran_ / GHR or G_αgust_ / GHR in the cardiac mucosa did not differ in the experimental groups vs. the control (Fig 5E). By contrast, in the pyloric mucosa, the administration of the Hp diet evoked an increase of the mean number of G_αtran_ / GHR-IR or G_αgust_ / GHR cells after 3 and 30 days vs. Ctr (Fig 5F).

There were no significant differences in the number of G_αtran_ / CCK-IR cells in the villi in the jejunum (Fig 5G), whereas there was a significant increased number of these cells in Hp3 (18.3 ± 2.8) compared to Ctr (14.5 ± 1.3, *P˂*0.05) in the crypts (Fig 5H). The number of G_αgust_ / CCK-IR cells in jejunal crypts was greater in Hp3 (16.8 ± 3.3) than Hp30 (10.8 ± 4.1), although this result did not reach statistical significance (*P* = 0.06) (Fig 5H).

Finally, in the large intestine, the quantitative analysis of G_αtran_ / or G_αgust_ / PYY-IR cells showed no difference in the three experimental groups (Fig 5I and 5J).

Regarding the percentages of expression of G_αtran_- or G_αgust-_IR in ECC subtypes, we observed statistical differences in the percentage of the colocalized G_αtran_- or G_αgust-_ 5-HT / total 5HT-IR cells in the duodenal villi and glands (S3 Table).

## Discussion

Physiological processes in the GI tract, such as secretomotor functions, digestion and absorption are coordinated and integrated events depending upon dietary intake and hormone release through constant monitoring of the luminal content by different sensory systems [1,4,6,36]. TASRs and taste-related molecules in the gut mucosa could serve as the initial molecular mechanisms underlying appropriate functional responses to luminal nutrients and non-nutrients contributing to gut chemosensitivity. This is supported by the localization of G_αtran_ and G_αgust_ in distinct populations of EECs in different mammals including rodents [13,20,23,37–39], pigs [27] and humans [24,40]. Here we showed that Hp diet affected the expression of the taste-related molecules, G_αtran_- or G_αgust_, expressed by EECs, which act as chemoreceptors in the GI tract [4]. The effects of a Hp diet on the density of G_αtran_- or G_αgust_-IR cells were more prominent at 30 days than at 3 days and particularly evident in the pyloric mucosa, compared to other regions of the gut. These findings expand previous observations on the effect of feeding and fasting and dietary factors, including low cholesterol and high fat diets, on the GI chemosensory system [27,28]. Several studies demonstrated that protein breakdown results in amino acids and protein-hydrolysates that activate sensory receptors in chemosensing EEC cells of the gastric mucosa, which modulate digestive functions including gastric emptying, acid and entero-pancreatic secretion and food intake, and contribute to the maintenance of energy homeostasis, via hormone (mainly peptides) secretion [5,36,41]. Recent evidence suggests that L-amino acids may be sensed by a group of G-protein coupled receptors which include TAS1R and TAS2R families, the calcium sensing receptor (CaSR) and the G-protein coupled receptor family C group 6 member A (GPRC6A) [42]. The CaSR mainly senses aromatic amino acids and calcium (Ca^2+^) [43–45], while the GPRC6A is a receptor that predominantly senses basic amino acids and Ca^2+^ and acts in concert with the CaSR [44,46].

Several studies have demonstrated that the G-proteins, G_αtran_- or G_αgust_, are signalling molecules transducing TAS1Rs and TAS2Rs functions [47–50], while CaSR and GPRC6A are transduced by Gαq-family proteins [51–53] or other Gαi-family proteins [54,55]. The increased density of G_αtran_ and G_αgust_-IR cells during Hp diet observed in this study might reflect the upregulation of TAS1Rs that included the TAS1R1, TAS1R2 and TAS1R3 subtypes that functions as dimers. The heteromeric combination of TAS1R1-TAS1R3 has been shown to function as a broad spectrum L-amino acid sensor, responsible for mediating perception of the savory “umami”, taste of monosodium glutamate [55,56], and responds to a wide variety of L-amino acids in the millimolar range [56]. On the other hand, the high content of proteins in the diet could enhance the expression of the sweet sensors, i.e. the TAS1R2 and TAS1R3, likely a compensatory effect of a decreased content of carbohydrates. This response appears to be in contrast to the TAS1R2 down-regulation induced by glucose administration reported in the mouse gut [57]. Since the degradation products of protein hydrolysis can be bitter, we cannot exclude that the Hp diets exert a modulatory role on the large TAS2Rs family [58] and therefore an increased amount of food-born bitter tastants (or, alternatively, increased amino acids per se) could result in increased TAS2R / G_αtran_ / G_αgust_ expression in the pyloric mucosa.

G_αtran_-IR cells density in pyloric mucosa was significantly increased after 3 days of the Hp diet administration, whereas the increase in G_αgust_-IR cells reached statistical significance only after 30 days of Hp diet. This suggests that the Hp diet evoked a differential regulation of the taste receptor system mainly involving G_αtran_ in the short-term and both G_αtran_ and G_αgust_ in the long-term. Our results indicated the occurrence of major changes in taste signaling molecules in the upper GI tract, mainly in the antrum, thus expanding previous data from our laboratory showing modulation of taste-related molecules and distinct TAS2Rs in the stomach following fasting and re-feeding [27] and in the upper small intestine with low-cholesterol diet [28]. Taken together these data indicate that different dietary manipulations affect taste signaling molecules and receptors throughout GI tract segments. Based on the "intestinal sensor cell hypothesis" [59], implying that nutrients can be sensed by EECs expressing TASRs, the stomach could be thought as the "first gate" monitoring food components and activating digestive processes or aversive responses in the case of potentially harmful substances [60]. This initial response would be followed by the functional response of the upper small intestine where digestion continues and absorption initiates.

Kinsey-Jones et al. (2015) [42] reported that a Hp diet was not effective in modulating GPRC6A expression in different regions of the mouse GI tract and hypothesized the presence of multiple overlapping systems mediating the effects of dietary amino acids and proteins. Our finding of changes in the expression of G proteins transducing taste receptors in certain regions of the gut in response to Hp diets are consistent with the notion of multiple receptors involvement for amino acid sensing in the gust as reported in the lingual epithelium [61,62].

The increased density of the overall GHR-IR cells population observed in the pyloric region in the Hp30 group is in line with the reported increase of GHR levels in plasma in rats and ruminant following long-term high-protein diets (7 days to 2 weeks) [63,64]. Furthermore, our observation of an increase in G_αtran-_ and G_αgust_-IR cells containing GHR-IR in the pyloric mucosa of Hp30 group compared to Hp3 and Ctr, is in agreement with recent findings showing that amino acids and di- / tripeptides are sensed by TAS1R1-TAS1R3, which stimulate a chemosensory signalling pathway regulating ghrelin release [35]. However, we did not see any quantitative change of GHR-IR cells after Hp3 diet, whereas Lejeune et al. (2006) [65] demonstrated that a four-day administration of a Hp diet resulted in suppression of GHR plasma levels. We did not see any change in the number of PYY-IR or PYY cell coexpressing G_αtran_ or G_αgust_-IR in the colon and rectum, though PYY release is increased by Hp diet stimulation in mammals, including humans [66–68]. These apparently discrepant results are likely due to the different measurements of GHR and PYY in tissue and blood and the different animal models. Whether an increased number of G_αtran_ and G_αgust_ / GHR-IR or G_αtran_ and G_αgust_ / PYY-IR cells is associated with increased circulating levels of these peptides was beyond the purpose of the present study and remains to be established.

Our finding that G_αtran_ and G_αgust_ cells in the small bowel co-expressed 5-HT extend previous data in the mouse [38]. Furthermore, the observation that G_αtran_ and G_αgust_ / 5-HT cells are increased in the Hp3 and Hp30 groups compared to controls, suggests that the effect of Hp diet on gut physiology (e.g. secreto-motor and nociceptive function) [69] involves the activation of the gut taste system via the release of 5-HT, a key signalling molecule in the gut.

The increase of the G_αtran_ / CCK cells in the jejunal crypts of Hp3 group might reflect activation of these cells by protein hydrolysates, peptides and amino acids, which have been reported to induce secretion of CCK by EECs expressing TAS1R1-TAS1R3 [70]. On the other hand, the decreased number of G_αtran_ / CCK cells after 30 days of high-proteins diet suggests that adaptive mechanisms come into play. CCK plays many roles in the digestive processes and has a well known inhibitory effect on food intake [71], effects that could be mediated by the activation of the taste receptor system in the gut.

In conclusion, this study shows that short- and, in particular, long-term Hp diet evoked selective changes in the expression of TASR related signalling molecules in subsets of EECs in different regions of the GI tract. Our findings further strengthen the hypothesis of a functional role of taste-related molecules in gut chemosensitivity and suggest a functional role of the gut taste system in nutrient-dependent—including proteins—gut functions.

## Supporting Information

S1 Fig Representative images of the pre-adsorption test of ghrelin (A), serotonin (5-HT, B) and peptide YY (PYY, C) primary antibodies.
(TIF)

S1 Table A Composition of experimental diets and energy density. * vitamins, minerals and amino acids integration
(DOCX)

S2 Table Body weight and feed consumption of experimental animals. Values are expressed as mean ± standard deviation.
(DOCX)

S3 Table Percentages of the colocalized G_αtran_- G_αgust_ -5-HT/ total 5HT-IR cells in the duodenal villi and glands. (DOCX)
