# Correction: Regulation of α-Transducin and α-Gustducin Expression by a High Protein Diet in the Pig Gastrointestinal Tract

**DOI:** 10.1371/journal.pone.0152647

**Published:** 2016-03-24

**Authors:** Roberto De Giorgio, Maurizio Mazzoni, Claudia Vallorani, Rocco Latorre, Cristiano Bombardi, Maria Laura Bacci, Monica Forni, Mirella Falconi, Catia Sternini, Paolo Clavenzani

There is an error in the Funding section. The correct funding information is as follows: The present work was supported by National Institutes of Health (NIH) grants DK098447 and 41301 (C.S.), grants from the Italian Ministry of Education, University and Research (MIUR; PRIN2009; R. De G.), from ‘Fondazione Del Monte di Bologna e Ravenna’, Italy, and funds from the University of Bologna (R. De G. and P.C.). The funders had no role in study design, data collection and analysis, decision to publish, or preparation of the manuscript.
