# Supplementary figures and images for: Regulation of α-Transducin and α-Gustducin Expression by a High Protein Diet in the Pig Gastrointestinal Tract
Source: PLoS One. 2016 Feb 12;11(2):e0148954. doi: 10.1371/journal.pone.0148954 (PMC4752509; doi:10.1371/journal.pone.0148954)

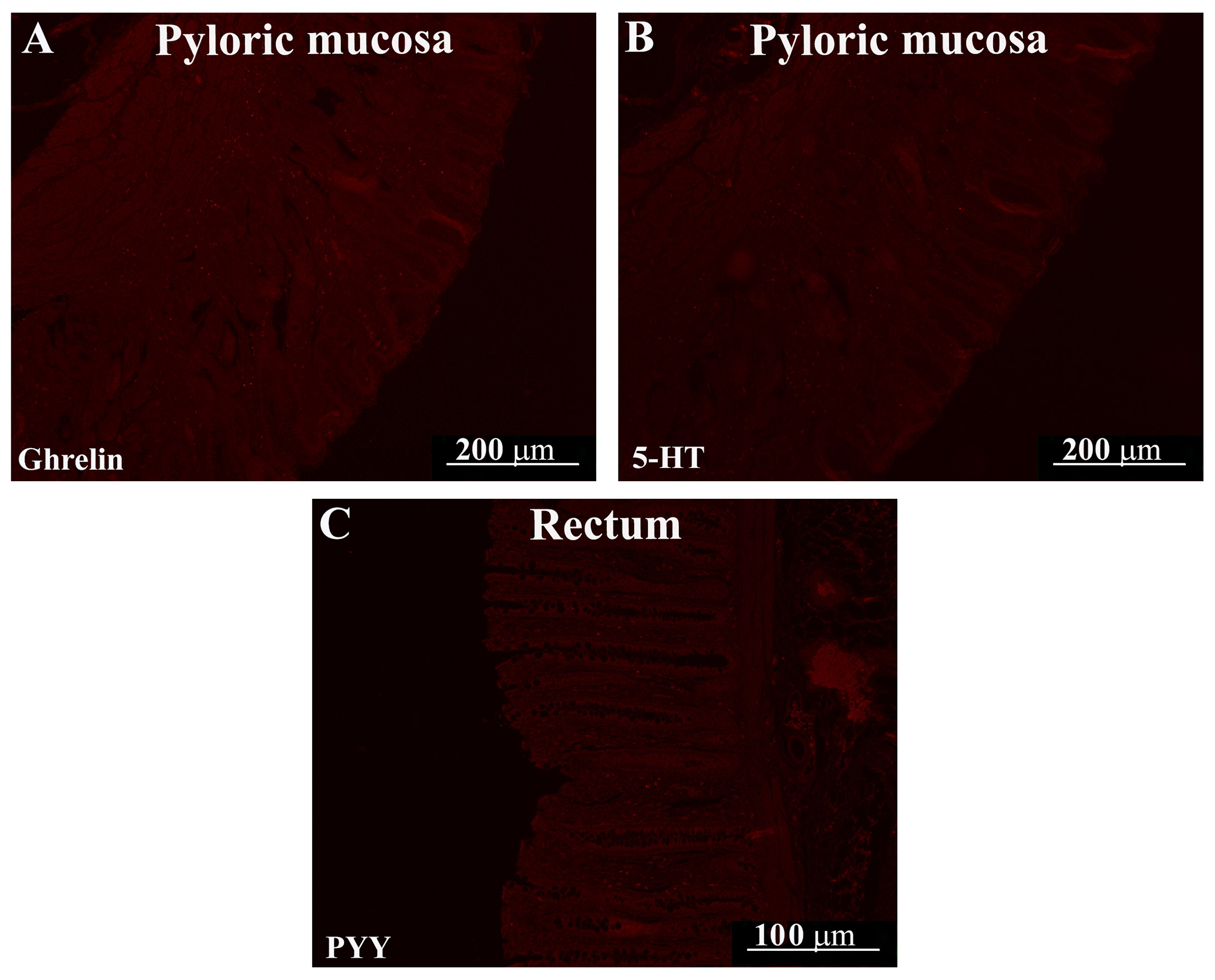

Supplement: S1 Fig — Representative images of the pre-adsorption test of ghrelin (A), serotonin (5-HT, B) and peptide YY (PYY, C) primary antibodies. (TIF) [file pone.0148954.s001.tif]
