# Supplementary material for: Regulation of α-Transducin and α-Gustducin Expression by a High Protein Diet in the Pig Gastrointestinal Tract
Source: PLoS One. 2016 Feb 12;11(2):e0148954. doi: 10.1371/journal.pone.0148954 (PMC4752509; doi:10.1371/journal.pone.0148954)
Supplement: S1 Table — * vitamins, minerals and amino acids integration (DOCX) [file pone.0148954.s002.docx]

| **Ingredient** |  | **Standard diet** | **High protein diet** |
| --- | --- | --- | --- |
| Maize meal | g/kg | 549 | 77 |
| Barley meal | “ | 110 | 250 |
| Wheat soft bran | “ | 150 | 200 |
| Soybean meal 44% | “ | 140 | 150 |
| Fish meal | “ | - | 150 |
| Potato protein | “ | - | 150 |
| Soybean oil | “ | 13 | - |
| Premix | “ | 38 | 23 |
| ***Composition*** |  |  |  |
| Moisture | g/kg | 112.0 | 110.8 |
| Crude protein | “ | 145.2 | 350.7 |
| Crude fibre | “ | 40.0 | 42.0 |
| Ether extract | “ | 42.0 | 35.0 |
| Starch | “ | 439 | 245 |
| Digestible energy | Kcal/Kg | 3284 | 3291 |
