# Supplementary material for: Regulation of α-Transducin and α-Gustducin Expression by a High Protein Diet in the Pig Gastrointestinal Tract
Source: PLoS One. 2016 Feb 12;11(2):e0148954. doi: 10.1371/journal.pone.0148954 (PMC4752509; doi:10.1371/journal.pone.0148954)
Supplement: S2 Table — Values are expressed as mean ± standard deviation. (DOCX) [file pone.0148954.s003.docx]

| **Body weight** |  | **Hp3** | **Hp30** | **Ctr** |
| --- | --- | --- | --- | --- |
| **Initial** | Kg | 43.20 + 2.1 | 42.60 + 1.8 | 43.10 + 1.6 |
| **Final** | Kg | 44.52 + 1.3 | 60.49 + 1.5 | 61.72 + 1.1 |
| **Feed consumption** | Kg/day | 1.4 ± 0.0 | 1.7 ± 0.2 | 1.7 ± 0.2 |
