# Supplementary material for: Regulation of α-Transducin and α-Gustducin Expression by a High Protein Diet in the Pig Gastrointestinal Tract
Source: PLoS One. 2016 Feb 12;11(2):e0148954. doi: 10.1371/journal.pone.0148954 (PMC4752509; doi:10.1371/journal.pone.0148954)
Supplement: S3 Table — (DOCX) [file pone.0148954.s004.docx]

| **Duodenum** | **G_αtran_/5HT (%)** | | | **G_αgust_/5HT (%)** | | |
| --- | --- | --- | --- | --- | --- | --- |
|  | **Ctr** | **Hp3** | **Hp30** | **Ctr** | **Hp3** | **Hp30** |
| **Villi** | 21.6 ^a^ | 19.8 ^a^ | 16.3 ^a^ | 59.5 ^b^ | 74.7 ^b^ | 69.8 ^b^ |
| **Glands** | 4 ^a^ | 8 ^a^ | 6.2 ^a^ | 55.9 ^b^ | 66.5 ^b^ | 55.7 ^b^ |

Different letters indicate a significant (*P*<0.05) statistical difference among groups.
